# When Nature plays upon an Ailment: A Case Report

**DOI:** 10.5005/jp-journals-10005-1135

**Published:** 2012-02-24

**Authors:** Rani Somani, Ripin Garewal, Poonam Preet Bhandari, Dilip Kumar

**Affiliations:** Professor and Head, Department of Pedodontics and Preventive Dentistry, DJ College of Dental Sciences and Research, Modinagar Uttar Pradesh, India; Postgraduate Student, Department of Pedodontics and Preventive Dentistry, DJ College of Dental Sciences and Research, Modinagar Uttar Pradesh, India; Reader, Department of Oral and Maxillofacial Surgery, DJ College of Dental Sciences and Research, Modinagar, Uttar Pradesh, India; Senior Consultant, Max Superspeciality Hospital, Saket, New Delhi India

**Keywords:** Radicular cyst, Odontogenic lesion, Surgical enucleation, Marsupialization, Mixed dentition

## Abstract

Of the most common odontogenic cysts, radicular cysts represent cystic lesions of inflammatory origin and are managed either by surgical enucleation or by marsupialization. This article aims to report a clinical case of radicular cyst of a huge proportion treated with a conservative management. An illustration of possible complete healing of such a cystic periapical lesion in mixed dentition with conservation of vital structures is covered.

**How to cite this article:** Somani R, Garewal R, Bhandari PP, Kumar D. When Nature plays upon an Ailment: A Case Report. Int J Clin Pediatr Dent 2012;5(1):61-63.

## INTRODUCTION

Radicular or residual cysts, also called as periapical cyst, are the most frequent odontogenic cysts of teeth bearing areas. Radicular cysts are rare in primary dentition,^1^ and represent only 0.5 to 3.3% of the total number of radicular cysts in both the primary and permanent dentition.^1,2^ These arise from epithelial Malassez rests in periodontal ligament as a result of inflammation. Most radicular cysts seen in the primary dentition are associated with mandibular molars.^3^

Marsupialization represents synonymous with Partsch’s operation,^4^ and is the conversion of a cyst into a pouch,^5^ it requires considerable aftercare and patient cooperation in keeping the cavity clean whilst it resolves and heals by relieving the internal pressure. This technique is indicated when cyst is in close proximity to vital structures and where there is significant risk of injury with enucleation. Several odontogenic developmental processes take place in a mixed dentition, hence, the surgical technique of choice should be the one with the least likelihood of iatrogenic damage. A case is presented for attempting the conservative treatment of odontogenic cyst and a workable protocol for this is applied.

## CASE REPORT

**Fig. 1:**
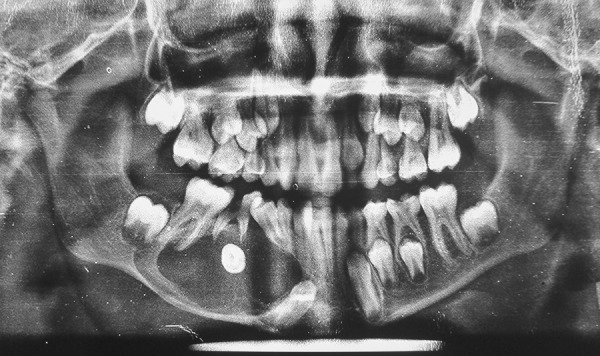
Preoperative panoramic radiograph showing well-defined periapical unilocular radiolucency

**Fig. 2:**
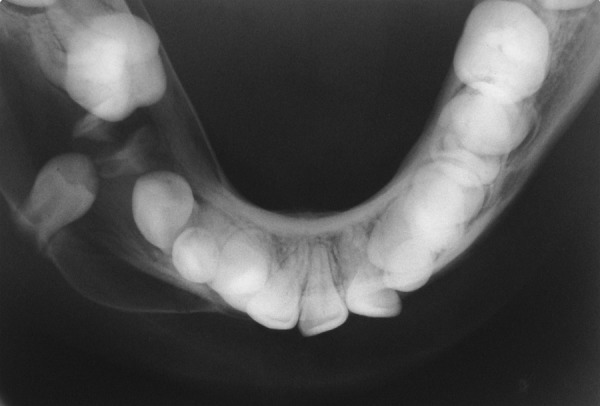
Occlusal radiograph showing expansion of buccal and lingual cortical plate

A 13-year-old male was reported to the Department of Pedodontics and Preventive Dentistry, DJ College of Dental Sciences and Research, Modinagar with a complaint of painless swelling in the right lower back tooth region since 2 months. Extraoral examination revealed a nontender and firm swelling on mandibular right side of face. Intraoral examination revealed a grossly decayed 85. Buccal vestibular expansion extending from distal aspect of 44 to mesial aspect of 46 was noticed. Panoramic radiograph revealed a single well-defined periapical unilocular radiolucency in relation to 85 region, with a radiopaque rim, of about 3 × 2 cm in size, extending from distal aspect of 42 to mesial root of 46 anteroposteriorly, involving tooth bud of 45 and displacing developing tooth bud of 43 inferiorly and mesially. The cyst caused tipping of the developing root of 44 and pushed it mesially causing the crown to incline distally (Fig. 1). Occlusal radiograph revealed a considerable expansion of buccal and lingual cortical plate of mandibular right side which was taken into account (Fig. 2). Based on history, clinical and radiographic examination, a provisional diagnosis of radicular cyst associated with 85 was made.

**Fig. 3:**
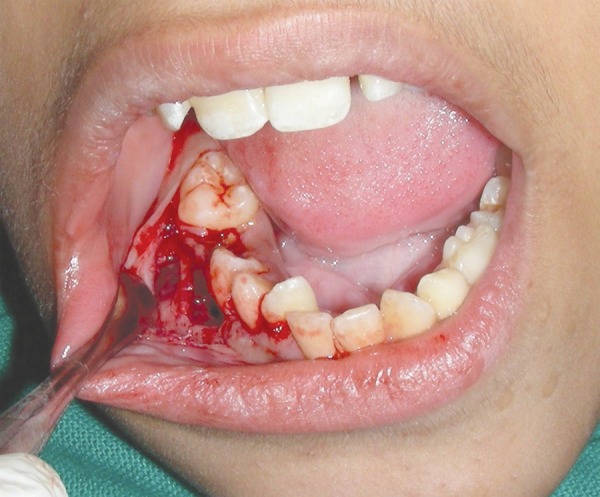
Flap reflected from 44 to 46 region to expose the cystic site

**Fig. 4:**
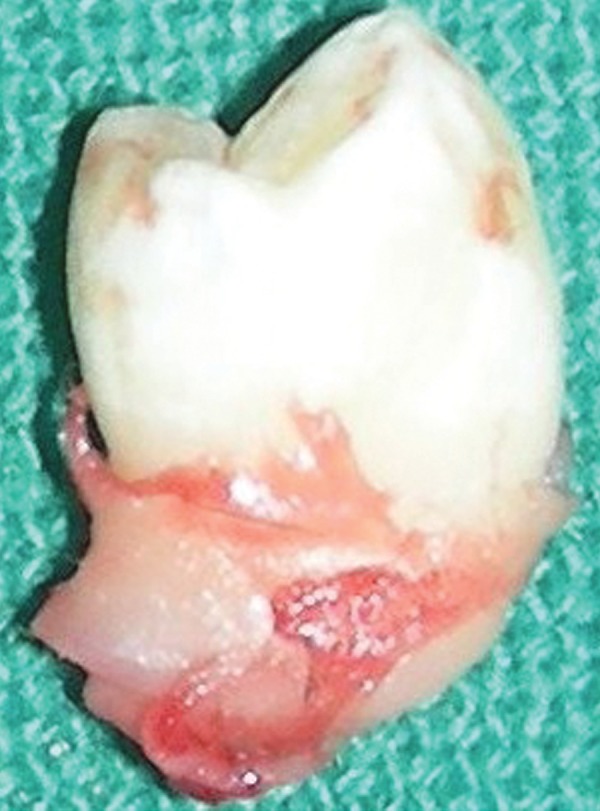
Extracted 85

**Fig. 5:**
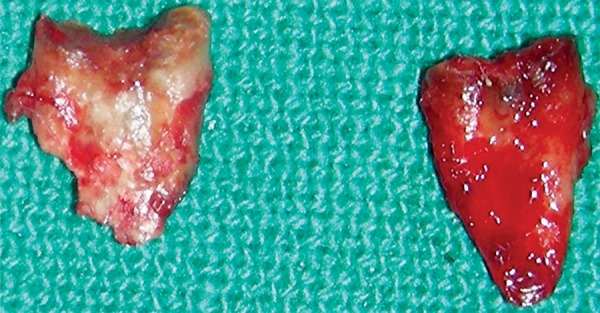
Removed developing tooth bud of 45

After the blood investigations and consent of the guardian of the patient, the case was posted for a surgical management of the lesion under local anesthesia. Crevicular incision was made from mesial of 44 to distal of 46 along the gingival margin and mucoperiosteal flap was raised. The socket margins were widened to gain access to the cystic cavity and cavity was decompressed (Fig. 3). The lingual cortical plate was left intact. Eighty-five was extracted (Fig. 4). Part of cystic lining was excised and developing tooth bud of 45 was removed based on operator’s understanding and expert opinions (Fig. 5). Wound irrigation was done with normal saline and excised cystic lining was sent for histopathology examination. The flap was sutured back with simple interrupted sutures. The cystic cavity remained exposed through extraction socket of 85.

**Fig. 6:**
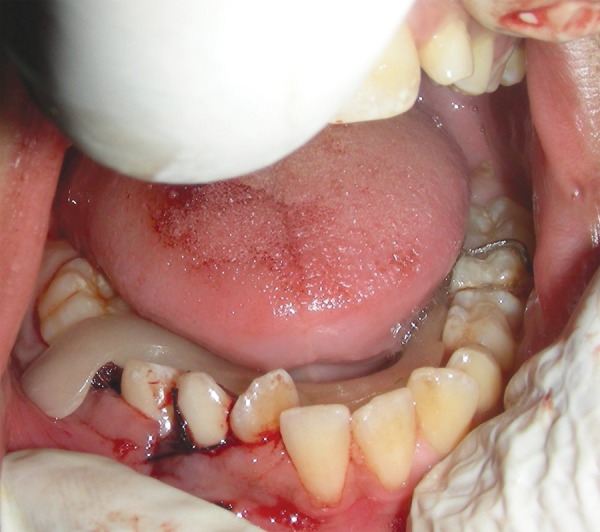
Obturator placed over surgical site

The surgical defect was packed with ribbon gauze soaked in betadine ointment. An obturator was prepared to promote bone healing, to prevent food accumulation and to maintain a patent surgical opening (Fig. 6).

**Fig. 7:**
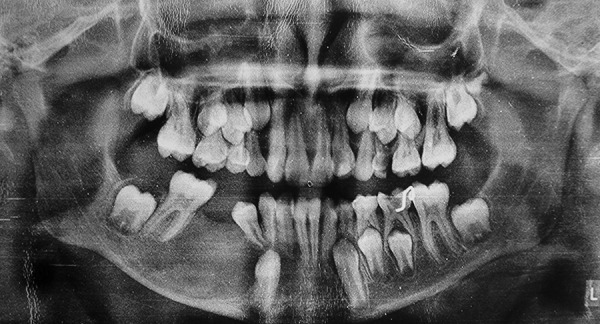
Postoperative panoramic X-ray at 6 months

Ribbon gauze dressing, dipped in betadine and glycerine to keep the cavity moist was replaced every alternate day for a period of 6 months. A bimonthly panoramic X-ray aided in keeping a track on the healing of site. A substantial reduction in the size of the cystic cavity as well as uprighting and promotion of eruption of 43 was seen in a 6 months follow-up panoramic X-ray (Fig. 7).

**Fig. 8:**
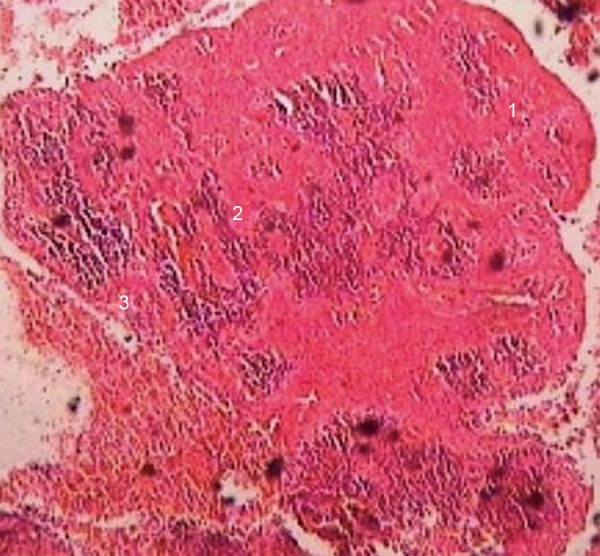
Histopathological view showing nonkeratinized stratified squamous epithelium (1), arcading pattern of proliferation (2) and dense chronic inflammatory cell infiltrate (3)

Histologically, a nonkeratinized stratified squamous epithelium, arcading pattern of proliferation and dense chronic inflammatory cell infiltrate were evident which were consistent with the diagnosis of radicular cyst (Fig. 8).

**Fig. 9:**
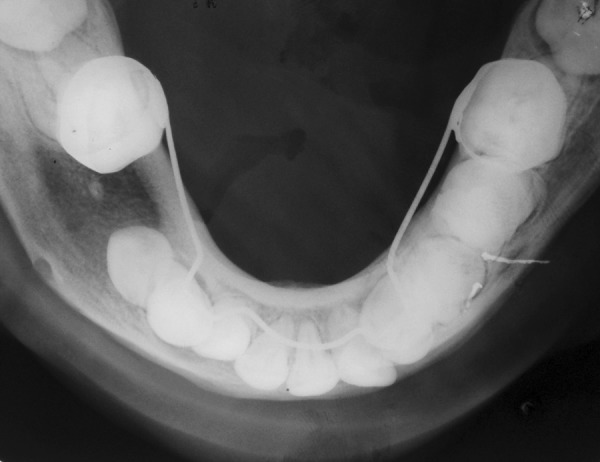
Lingual arch space maintainer placed

A lingual arch space maintainer was planned and placed to prevent mesial migration of 46 and to promote eruption of 44 into normal occlusion (Fig. 9).

## DISCUSSION

Cysts are usually enucleated, where the cystic lining is separated from its inner bony surface and removed and cavity is allowed to fill with blood clot. However, a cyst can be marsupialized to relieve the internal pressure as well.

In our case, the marsupialization technique applied with extraction of associated primary tooth and developing permanent tooth bud of 45 and preservation and promotion of erupting tooth bud of 43 appeared to be the most suitable treatment option due to lower morbidity and faster healing process, with a healing period of 6 months to our first phase of treatment. Placement of a lingual arch fixed space maintainer was our second phase of treatment.

According to Neaverth,^4,6^ marsupialization consists of deroofing the outer wall of a cyst by surgical incision and establishing a permanent opening by suturing the remaining cystic wall to the mucosal surface followed by an obturator treatment.

The main intention of obturator placement was to promote healing of the wound, followed by placement of lingual arch to prevent migration of 46 and provide ample space for 44 to erupt in normal occlusion.

## CONCLUSION

Marsupialization is favored because of lower morbidity and the fact that bony ingrowth occurs as the lesion shrinks in size, resulting in more normal bony contour. It is very likely that reduction of intracystic pressure is a key factor in the healing process. Although it is not known what percentage of radicular cyst can be expected to heal with only marsupialization, it is a viable treatment modality that bears consideration when treating large cystic lesions.^7^An operator should maintain constriction with conservation of vital structures as much as he can when operating such cases.

## References

[1]  Shear M (1992). Radicular and residual cysts. In: Cysts of the oral region..

[2]  Bhaskar SN (1966). Periapical lesion: Types, incidence and clinical features.. Oral Surg Oral Med Oral Pathol.

[3]  Mass E, Kalpan I, Hishberg A (1995). A clinical and histopathological study of radicular cysts associated with primary molars.. J Oral Pathol Med.

[4]  Neaverth EJ, Burg HA (1982). Decompression of large periapical cystic lesions.. J Endodont.

[5]  Sakkas N, Shoen R, Schulze D, Otten JE, Schmelzeisen R (2007). Obturator after marsupialization of a recurrence of a radicular cyst of the mandible.. Oral Surg Oral Med Oral Pathol Oral Radiol Endod.

[6]  Takase T, Wada M, Nagahama F, Yamazaki M (1996). Treatment of large radicular cysts by modified marsupialization.. J Nichon Univ Sch Dent.

[7]  Riachi F, Tabarani C (2010). Effective management of large radicular cysts using surgical enucleation vs marsupialization.. IAJD.

